# How Does Human Capital Spillover Inflow of Foreign Workers Affect Economic Growth?

**DOI:** 10.3389/fsoc.2021.750946

**Published:** 2021-12-22

**Authors:** Etty Puji Lestari, Caroline Caroline

**Affiliations:** ^1^ Department of Economics, Faculty of Economics, Universitas Terbuka, Tangerang Selatan, Indonesia; ^2^ Department of Economics, Faculty of Economics and Social Science, Universitas Sultan Fatah, Demak, Indonesia

**Keywords:** foreign worker, spatial autoregressive model, human capital, spillover, economic growth

## Abstract

The ASEAN Economic Community provides opportunities for foreign workers to enter Indonesia, including Central Java Province. The existence of these foreign workers tends to negatively and positively impact the regional economic growth of the country. Therefore, this study aims to analyze the effects of foreign workers’ human capital spillover inflow on the economic growth of Central Java. The Euclidean distance spatial weight matrix was used to calculate the spatial autoregressive model from 2015 to 2020. These results indicate that the presence of skilled foreign workers positively impacts increasing economic growth in Central Java Province. The influx of foreign workers, along with the influx of investment, encourages local workers to follow the performance of foreign workers. This study suggests a policy to encourage technology transfer from foreign workers to local workers. The government is also expected to strengthen local workers’ productivity to compete with foreign workers.

## Introduction

The AEC implementation provides opportunities for highly educated foreign workers with special skills to migrate to Indonesia (Chia, 2014; Adhariani, 2020; Elms, 2020; Ho, 2019), including Central Java Province. This led to a 7% increase in the economic growth of this region, thereby making it an attractive place for foreigners to work (BPS Central Java, 2021). Furthermore, Central Java Province is one of the favorite provinces for investment due to accessible licensing services (Windhyastiti et al., 2020).

The free trade implementation in ASEAN regions has positive and negative impacts on labor (Chirathivat, 2002; Chin and Stubbs, 2011). One of the advantages is that many foreign capitals are invested in Indonesia, thereby creating job opportunities. Furthermore, the domestic regulation of goods and services tends to spin quickly, while export activities are efficiently executed. The negative impact is when goods and services are easily imported into the country, thereby threatening the domestic economy. This also led to the ease of labor both internally and externally. Therefore, the government needs to analyze the Indonesian human resources in the face of free trade.

Some studies on human capital spillover and externalities related to economic growth were carried out by Lucas (1988) and Romer (1990). These were reviewed in the form of physical capital and uneducated labor in endogenous growth models. Benhabib and Spiegel used a traditional accounting approach to evaluate the contribution of human capital to aggregate growth (Benhabib & Spiegel, 1994). This was demonstrated using total factor productivity, which led to the existence of world leaders in technology or the catch-up effect that eventually resulted in endogenous growth impacts. The studies carried out by Romer (1990), Benhabib & Spiegel (1994), and Brunow & Hirte (2009) used spatial error models (SEM) to determine workers’ age patterns with respect to the regional average productivity.

Caroline et al. (2019) used the Euclidean distance spatial weight matrix to calculate SAR. Fixed-effects SAR showed significant capital and spatial lag variables, implying a 0.6% growth spillover in ASEAN countries. However, ASEAN member countries grow by relatively 1%, and their economy increases by 0.6%, with a capital coefficient of 3.67e-12 at a significant rate of *α* = 1%. This is influenced by the value of capital, UWS, labor, and spatial residuals of the other 10 member countries (Indonesia, Malaysia, Brunei, Cambodia, Thailand, Singapore, the Philippines, Laos, Myanmar, and Vietnam) with similar characteristics. Another study carried out by Caroline et al. (2020) stated that the spillover of local labor in Central Java Province was realized through many foreign investment (PMA) projects established in districts and cities.

The studies carried out by Caroline et al. (2020) and Ho et al. (2013) adopted the Solow growth model developed by Mankiw based on the role of education in improving economic growth. Education tends to boost economic growth through labor productivity. Therefore, foreign workers who migrate to Central Java Province are expected to be highly disciplined and exhibit good work ethics, which leads to competitive labor with the locals. Furthermore, this study analyzes the impact of the inflow of foreign workers to the Central Java Province using the Euclidean distance spatial weight matrix.

## Literature Review

### Human Capital Spillover and Economic Growth

Spillover is described as the externality of economic activity that affects the parties involved. According to Fleisher et al. (2008), it is the interaction between human capital and the gap or distance per capita income of a region compared to an advanced place. The model designed by Fleisher et al. (2008) is in accordance with the research carried out by Nelson and Phelps (1966) on technological diffusion. They introduced the first model, which was related to the importance of technological advances. It was proposed based on the assumption that technological advances were inherent in labor augmenting, otherwise known as “Harrod-neutral.”

Y(t)=F[K(t), A(t)L(t)], (1)

where Y(t)  is the output, K(t) is capital, L(t) is labor, A(t) is the technology index, and  t is time. Nelson and Phelps (1966) realized that technological advances depend on human capital (h) and technological gap [T(t)] , as stated in

A(t)=φ(h)[T(t)-A(t)] (2)

or equivalent to

$$\frac{\;\overset{\cdot }{A}\left(t\right)}{A\left(t\right)}=\varphi \left(h\right)\left[\frac{T\left(t\right)-A\left(t\right)}{A\left(t\right)}\right],\;\phi \left(0\right)=0,\;\phi^{\prime \left(h\right)}>0.$$ (3)

Eqs. 2, 3 indicate the level of technological gap depending on the human capital, through the function φ(h) , where $\phi^{\prime \left(h\right)}>0$ . The ϕ(h) function also represents the ability to adapt and adapt to the latest technologies. In theory, the level of knowledge is derived from its exponential rate; therefore, $T\left(t\right)=T\left(0\right)e^{\mathrm{\lambda t}}$ . The implications of the growth model designed by Nelson and Phelps (1966) are 1) in the short term, technological progress $\left[\frac{\overset{\cdot }{A}\;\left(t\right)}{A\left(t\right)}\right]$ is influenced by human capital (h) and 2) in the long term, technological progress needs to be consistent with its exponential rate (λ) . It consistently showed that the existence of human capital greatly influenced technological catch-up and diffusion.

Furthermore, Benhabib and Spiegel (1994) developed a growth model similar to Nelson and Phelps, (1966) by including domestic innovation. They assumed that every period reports the existence of several countries as world leaders in technology. Nelson and Phelps’s growth model, modified by Benhabib and Spiegel (1994), is written as follows:

$$\left[\frac{\overset{\cdot }{A}\;\left(t\right)}{A\left(t\right)}=g\left(h_{i}\right)+\varphi \left(h_{i}\right)\left[\frac{\mathrm{ma}x_{j}A_{j}\left(t\right)-A_{i}\left(t\right)}{A_{i}\left(t\right)}\right]\right],\;\mathrm{i=1,\ldots}..\mathrm{,n,}$$ (4)

where $g\left(h_{i}\right)$ is an endogenous growth rate that represents “domestic innovation,” $\varphi \left(h_{i}\right)\left[\frac{\mathrm{ma}x_{j}A_{j}\left(t\right)-A_{i}\left(t\right)}{A_{i}\left(t\right)}\right]$ represents technological diffusion, and $\mathrm{ma}x_{j}A_{j}\left(t\right)$ is the maximum technology index in one or more regions. Human capital $\left(h_{i}\right)$ uses “years of schooling” and “educational attainment” proxies in accordance with the growth model developed by Benhabib and Spiegel (1994) and Nelson and Phelps (1966). In addition, Benhabib and Spiegel (1994) adopted the total factor productivity (TFP) variable as a proxy for the growth of technological advances. Based on their research, human capital not only encourages a country’s ability to adapt and adapt to new technologies, rather it also involves the ability to develop technological innovation domestically.

Fleisher et al. (2008) modified both models designed by Benhabib and Spiegel (1994) and Nelson and Phelps (1966) by ensuring that the per capita and distance income represent the technological gap. This led to the modification of the technological progress growth as stated in Equation 5

$$\frac{\dot{A}\left(t\right)}{A\left(t\right)}=h_{i}\left[\frac{1}{d_{\max\_i}}\left(\frac{y_{\mathrm{max,t}}-y_{\mathrm{it}}}{y_{\mathrm{it}}}\right)\right],$$ (5)

where $h_{i}$ is the proportion of the population that attained the minimum university education in region i, $y_{\mathrm{it}}$ is GDP per capita in the same area and time *t*, $y_{max_{t}}$ is the highest GDP per capita at time *t*, and $d_{\max\_i}$ is the distance between country *i* and nations that have the highest GDP per capita. Fleisher et al. (2008) further represented the function $h_{i}\left[\frac{1}{d_{\max\_i}}\left(\frac{y_{\mathrm{max,t}}-y_{\mathrm{it}}}{y_{\mathrm{it}}}\right)\right]$ as the “human capital spillovers.” For instance, assuming $y_{\mathrm{max,i}}$ **=** $y_{\mathrm{it}}$ , then the spillover value is zero (spillover term = 0).

### Determinant of Labor Migration

Factors affecting labor migration include age, family factors, education, distance, unemployment rate, and government policies. According to Sjaastad (1962), one of the factors of labor migration to other regions is when the worker is older than 18 years and is searching for a job that meets the criteria and demands of the workforce after graduation. However, some decide to continue schooling.

In terms of family factors, a study carried out by Mincer (1978) reported that unmarried workers in terms of marital status tend to migrate to other regions in search of a decent job, while the reverse is the case for couples. In terms of education, Juhn et al. (1993) reported that highly educated workers are bound to migrate to other regions; besides, their experiences have a positive impact on the demand for labor. Davies et al. (2001) reported that distance is a labor consideration for migration. In addition, the workforce has potential to obtain information related to employment opportunities, and the further the distance, the higher the transportation cost. Meanwhile, when viewed from the policy aspect, Green and Hendershott (2001) and Borjas (2006) reported that the high level of international immigration into an area reduces and simultaneously increases incoming and outbound migration, respectively.

## Research Methods

An explanatory spatial data analysis (ESDA) approach and Euclidean distance spatial weight matrix were used to calculate the spatial autoregressive model. This research analyzed data acquired in 29 regencies and six cities in Central Java from 2015 to 2020. The variable descriptions used in this study are shown in Table 1.

**TABLE 1:** Variable description.

No.	Variable	Indicator	Unit	Sources
**1**	Economic growth of regency or city	PDB per capita	IDR	BPS Central Java
**2**	Capital stock	Domestic fixed capital formation (PMTB)	IDR	BPS Central Java
**3**	Human capital spillover	—	—	—
—	a. The average duration of a school (AS)	The average duration of a school	Year	BPS Central Java
—	b. Local workers (LW)	Residents aged 15 and above who have worked for the past week according to their educational qualification and those who have not yet attained university in the country	Person	BPS Central Java
—	c. Foreign workers (FW)	Residents aged 15 years and older who have worked for the past week according to their educational qualification, those who have not yet attained university, and those who migrated to the Province of Central Java	Person	Pusdatin Kemnaker

The global Moran’s index was adopted to analyze the presence or absence of spatial autocorrelation in the data. It is also used to identify spatial interactions for the entirety of observation with its range of values in accordance with a standardized spatial weight matrix of −1 ≤I ≤1 . The values −1 ≤I ≤0 and 0 ≤I ≤1 indicate negative and positive spatial autocorrelation, respectively. The zero-value global Moran index implies that it is not in groups (see Table 2).

**TABLE 2:** Feature patterns formed from Global Moran’s I.

Index Moran Global	Explanation
I > 0 and positive	Spatial interaction patterns cluster
I > 0 and negative	Patterns of diffuse or divergent spatial interactions
I < 0	Patterns of random spatial interaction
I = 0	Spatial interaction patterns spread due to high and low feature values in the data set

Sources: Caroline et al. (2020), Tiefelsdog (2002), and Tiefelsdolfand and Boots (1997).

Spatial autocorrelation formed using the global Moran’s I method only reads either positive or negative coefficient value marks. The positive mark implies the spatial clustering of similar values, while that of the negative identifies the existence of checkerboard patterns. Global Moran’s I value is assumed to be significant depending on the distribution of its statistical tests. This is based on two methods, namely, the random permutation test and the distribution approach. Global Moran’s I coefficient values that are greater and less than the expectations of −1/(n-1) are indicative of positive and negative spatial autocorrelation, respectively (Fischer and Wang, 2011).

ESDA is a Euclidean distance spatial weight matrix used to explore data and calculate the SAR model. The first step in ESDA is to calculate the Euclidean distance spatial weight matrix using GeoDa version 1.18 application to determine the coordinates *x* and *y* of an entity. The second step is to obtain the spatial autoregressive (SAR) model using SPSS 15. This is carried out by calculating the ordinary least square (OLS) to determine the parameter that affects the economic growth of Central Java, after which the likelihood ratio (LR) test and Hausman Test are used to ascertain whether to use the fixed or random effect. The results are known to use a spatial autoregressive model (SAR) with a fixed effect as follows:

$$PDB{\;}_{it}=\rho \sum_{j=1}^{n}W_{ij}PDB_{jt}+\beta_{1}CAP_{it-1}+\;\beta_{2}AS_{it-1}+\;\beta_{3}LW_{it-1}+\;\beta_{4}FW_{it-1}+\;\varepsilon_{i},$$

where

i = 1,...,n,

i = observed districts/cities,
j = other districts/cities,

ρ = i≠j ,
W = matrix of spatial weights with Euclidean distance approach,

β = regression coefficient,
CAP = capital,
AS = the average duration of a school,
LW = local worker,
FW = foreign worker, and

$\varepsilon_{i}\;$ = error term.

## Results and Discussion

### Euclidean Distance Spatial Weight Matrix

This study applied the Euclidean distance weight matrix to ensure that the calculated results correlate with the geographical distances. The value obtained from the Central Java shp Map was processed with GeoDa software to obtain the *x* and *y* coordinates, whereby 1 Euclidean distance is equivalent to 15.91 km.

Table 3 shows the spatial weight matrix with Euclidean distance realized using *x* and *y* coordinates from 29 districts and six cities in Central Java Province. This helps solve proximity and time issues, including labor and information mobility. According to Table 3, the Semarang regency is at *x* 109.12 and *y* −6.87 coordinates. Meanwhile, Semarang city and Demak regency are located at *x* 110.47 and *y* −7.27 and *x* 110.82 and *y* −7.56 coordinates, respectively.

**TABLE 3:** Spatial weight matrix with Euclidean distance.

No.	Regency/city	Coordinate points *x*	Coordinate points *y*	No	Regency/city	Coordinate points *x*	Coordinate points *y*
1	Regency Cilacap	108.89	−7.49	19	Regency Kudus	109.68	−6.89
2	Regency Banyumas	110.63	−6.91	20	Regency Jepara	110.39	−7.02
3	Regency Purbalingga	110.93	−7.12	21	Regency Demak	110.82	−7.56
4	Regency Banjarnegara	109.66	−7.35	22	Regency Semarang	109.12	−6.87
5	Regency Kebumen	109.18	−7.46	23	Regency Temanggung	109.62	−7.06
6	Regency Purworejo	109.86	−7.02	24	Regency Kendal	109.4	−7.04
7	Regency Wonosobo	111.39	−7.07	25	Regency Batang	109.4	−7.04
8	Regency Magelang	110.65	−7.42	26	Regency Pekalongan	109.12	−7.03
9	Regency Boyolali	108.93	−7.06	27	Regency Pemalang	110.14	−7.06
10	Regency Klaten	110.22	−7.48	28	Regency Tegal	110.99	−7.26
11	Regency Sukoharjo	110.77	−6.55	29	Regency Brebes	109.91	−7.42
12	Regency Wonogiri	111.02	−7.66	30	Magelang city	109.41	−7.32
13	Regency Karanganyar	109.62	−7.65	31	Surakarta city	109.97	−7.71
14	Regency Sragen	110.16	−7.04	32	Salatiga city	111.46	−6.78
15	Regency Grobogan	110.62	−7.69	33	Semarang city	110.47	−7.27
16	Regency Blora	110.25	−7.50	34	Pekalongan city	110.97	−7.39
17	Regency Rembang	111.04	−6.74	35	Tegal city	110.83	−7.68
18	Regency Pati	110.50	−7.74	—	—	—	—

Source: data processed with GeoDa.

### Global Moran’s I Calculation Results

The following Table 4 shows that global Moran’s I coefficient values are negative and insignificant, meaning that the workforce migrating into Central Java Province spreads across several districts and cities.

a) This study used confidence and significance levels of *α* = 5%.
b) Moran’s global index was significantly used to identify both similar and dissimilar spatial interaction patterns within groups.
c) Positive and negative patterns indicate the presence of convergent or clusters and a spread or divergence, respectively.
d) Moran’s global index values were not significantly used to identify the absence of similar and dissimilar spatial interaction patterns within groups.

(b), (c), and (d) were adopted from the research carried out by Anselin (1995).

**TABLE 4:** Global Moran’s I.

**Year**	**Migrant worker**
2015	−0.021
2016	−0.029
2017	−0.029
2018	−0.030
2019	−0.030
2020	−0.029

Source: data processed with Stata.

Table 5 shows that in 2015, 901 foreign workers migrated to Central Java Province, whereas in 2020, the number increased to 2,905, with 1,211, 450, and 212 recorded in Banyumas regency, Semarang city, and Jepara, respectively.

**TABLE 5:** Number of foreign workers in regencies/cities in 2015 and in 2020.

No.	Regency or city	Foreign workers 2015	Foreign workers 2020	No.	Regency or city	Foreign workers 2015	Foreign workers 2020
1	Regency Cilacap	19	34	19	Regency Kudus	1	4
2	Regency Banyumas	2	1,211	20	Regency Jepara	126	212
3	Regency Purbalingga	63	9	21	Regency Demak	5	29
4	Regency Banjarnegara	2	5	22	Regency Semarang	69	98
5	Regency Kebumen	1	1	23	Regency Temanggung	20	34
6	Regency Purworejo	1	1	24	Regency Kendal	49	51
7	Regency Wonosobo	2	1	25	Regency Batang	82	39
8	Regency Magelang	1	6	26	Regency Pekalongan	2	5
9	Regency Boyolali	36	58	27	Regency Pemalang	5	1
10	Regency Klaten	35	35	28	Regency Tegal	6	11
11	Regency Sukoharjo	126	171	29	Regency Brebes	8	18
12	Regency Wonogiri	4	16	30	Magelang city	1	0
13	Regency Karanganyar	1	10	31	Surakarta city	1	171
14	Regency Sragen	1	13	32	Salatiga city	16	65
15	Regency Grobogan	8	148	33	Semarang city	196	450
16	Regency Blora	2	1	34	Pekalongan city	3	5
17	Regency Rembang	1	1	35	Tegal city	1	11
18	Regency Pati	5	9	—	—	—	—

Source: data processed with GeoDa.

Before verifying the spatial regression model of the panel data, a classic test known as the goodness of fit was performed. The next step is to test the fixed or error effect using the LR test. Table 6 shows the results of the classic pooled OLS regression calculation. Variables with a positive and significant influence on GDP per capita are referred to as capital.

**TABLE 6:** Regression calculation result with OLS Pooled method.

**Variable**	**Coefficient**	**t-statistic**	***P*>|t|**
Constanta	15.93^*^	40.09	0.00
CAP	4.2e-07^*^	12.33	0.00
AS	0,03	0.80	0.42
LW	−4.56e-06	−0.17	0.86
FW	1.56e-06	0.13	0.89
Number of observations		—	175
R squared	—	—	0.48
Coef F statistic	—	—	40.58^*^
Prob F	—	—	0.00

Source: processed data.

Description: dependent variable GDP per capita; ^*^significant at the level of 5 percent.

The OLS pooled method is used to validate the equation or economic growth model. A significant value indicates that *α* = 5% with a coefficient of 40.58. Free variables that affect economic growth are Constanta and capital with a significant rate of *α* = 5%. Based on the OLS pooled testing, only capital variables have positive and significant results on economic growth. Meanwhile, the reverse is the case for the number of educated local workers (Table 7).

**TABLE 7:** Likelihood ratio (LR) test.

**Model**	**Chi-square**	***P*-value**
SAR fixed effect	43.02	0.00
SAR random effect	−52.13	1.00

Source: processed data.

The level of confidence used in this study was *α* = 5%.

Furthermore, an LR test was carried out to determine whether or not the model used fixed or random effects. Table 8 shows that the spatial autoregressive model was realized with a fixed effect obtained using the LR test at a significant level of *α* = 5%.

**TABLE 8:** Estimated fixed-effects SAR parameters.

Independent variable	SAR fixed effect	
	Coefficient	Z-table
Constanta	15.78	13.47
Capital	2.67e-07	4.85
AS	−0.0151	−0.27
LW	1.50e-06	4.96
FW	−2.22e-06	0.87
Spatial rho	0.34	8.37

Source: data processed, 2021.

The results of the SAR state that the presence of foreign workers has a positive impact on economic growth. The number of foreign workers who enter along with the entry of investment provides a spur for local workers to follow the performance of foreign workers. Foreign workers brought to Central Java Province are educated, skilled workers. This is expected to provide technology transfer to local workers (Erliz Nindi Pratiwi, 2013; Pratiwi and Mahmudah, 2013). The results of the SAR show that the presence of foreign workers can encourage the economy through the given performance. Many foreign workers brought along with the investment can drive the local workforce to improve their performance.

Meanwhile, the analysis results for local workers show that the presence of local workers has not had an encouraging impact on economic growth. This is due to the large number of local workers who work in sectors that do not have high skills. Most investors who invest in Central Java Province already include skilled labor, so they involve only a small local workforce.

Fixed-effects SAR results indicate that the capital and spatial lag variable are significant. This implies a growth spillover of 0.34%. However, assuming the average regency or neighboring city in Central Java develops by relatively 1%, its growth is boosted by 0.34%, with a capital coefficient and significant rate of 2.67e-07 and 5%, respectively. The spatial interaction and effects were obtained from each region in Central Java Province.

The spatial coefficient lag (*δ*) or rho 0.99283791 indicates the magnitude of the interaction between the regency or city’s GDP per capita value and neighboring regions. Meanwhile, the coefficient of 2.67e-07 indicates a 1% increase in capital and GDP per capita. Spatial rho 6 percent means that assuming Central Java Province grows by 3 percent. It is bound to affect the 29 regencies and six cities, with a productivity correlation of 1.5 percent. Therefore, the average number of foreign workers contributes to Central Java’s economic growth. Economic growth increases the value of goods and services and capital formation, which supports production (Kanu & Ozurumba, 2014). Therefore, an increase triggers the potential for a rise in production activities. Capital formation is one of the essential determinants for improving economic growth (Amri & Aimon., 2017; Kanu & Ozurumba, 2014).

The increase in capital stocks is due to the relationship between capital formation and economic growth, which supports production activities, as illustrated in the Solow growth theory (Ho et al., 2013; Solow, 1994). Its existence drives economic growth in Central Java.

**FIGURE 1:**
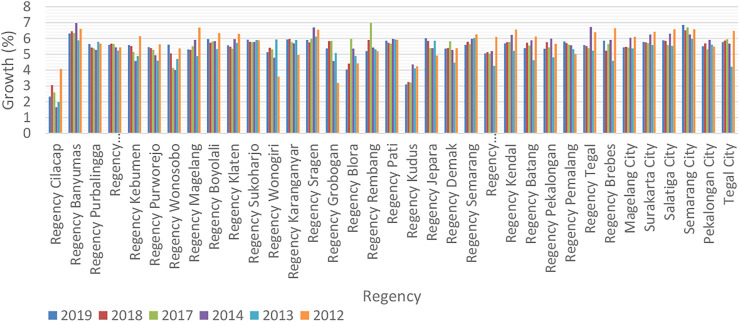
Economic growth in regency/city in Central Java.

Figure 1 describes the economic growth of various cities in Central Java Province before and after the AEC implementation, presumed to be triggered by capital. The study carried out by Intisar et al. (2020) also stated that human capital and economic growth have unidirectional relationships. It was further discovered that human capital and foreign direct investment accelerated trade openness.

**FIGURE 2:**
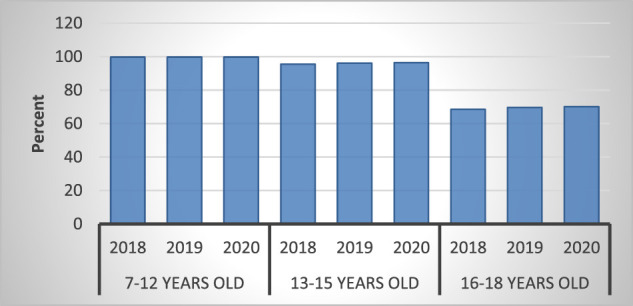
School enrollment rate.

Meanwhile, the average number of educated foreign workers has a positive and insignificant influence. These findings show that an excellent educational level aids in securing employment opportunities, both in the formal and informal sectors with a limited population of well-educated workers. The data show that the level of education attained by the average working age is between 60 and 70 % (BPS Central Java, 2021). This means that many of the workers lack appropriate education, as shown in Figure 2.

The impact of human capital spillover on the economic growth of Central Java Province has been influenced by the inflow of foreign workers since the implementation of the ASEAN Economic Community in 2015. Table 9 shows the inflow of foreign workers in Central Java Province as of December 31, 2020. A total of 12,311 were recorded, with 10,487 working in cross-province, while 1,640 and 184 working in Central Java and cross-regency, respectively.

**TABLE 9:** Inflow of foreign workers in Central Java Province, December 31, 2020.

**Inflow of foreign workers**	**Total**
Cross-province	10,487
Cross-regency/city in the province	184
Only one regency/city in the province	1,640
Total	12,311

Source: Disnakertrans Central Java Province, 2021.

**FIGURE 3:**
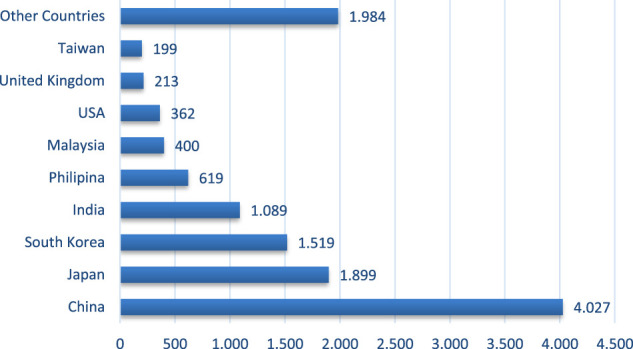
Inflow of foreign workers in Central Java Province, 2020. Source: Disnakertrans Central Java Province, 2021.

Figure 3 shows the inflow of foreign workers in Central Java Province. The foreign workers are mainly from the PRC, followed by Japan, South Korea, and India. China’s increased workforce is due to increased investment from the country. Thus, more and more experts accompany the investment. The local government hopes that there will be a transfer of technology from foreign labor to local labor. According to the rules, every foreign worker must be accompanied by local workers. So in the future, there will be many local workers who master the skill.

The impact of human capital spillover on the economic growth of Central Java Province is influenced by a large number of foreign workers in various fields. According to Table 10, 4,559 foreign workers have professional jobs, followed by managers, consultants, directors, and commissioners, respectively. One of the causes of the number of skilled foreign workers entering Central Java is because investors usually bring technology and workers into Indonesia. The local government states that the labor allowed to enter is specific skilled labor.

**TABLE 10:** Number of foreign workers by occupation Central Java Province, 2020.

No.	Foreign workers by occupation	Total
1	Professional	4,559
2	Manager	3,998
3	Consultant	3,330
4	Directors	994
5	Technician	253
6	Supervisor	249
7	Commissioner	88

Source: Disnakertrans Central Java Province, 2021.

Influxes of foreign workers in this province are experts in various fields, and this serves as a chain of investment packages. Table 11 shows a total of 961 projects that were realized in 2019 with an investment value of 1,509,660.70 thousand $. The influx of investment mainly originates from transportation, warehouse, telecommunications, electricity, gas, and water sectors located in Tegal, Semarang, Cilacap, Grobogan, and Kudus. Simultaneously, these projects are found in Batang, Jepara, Semarang, Brebes, and Kendal. The presence of foreign workers encourages the transfer of knowledge to the local ones, thereby aiding them to work more effectively and efficiently.

**TABLE 11:** Realization of foreign investment project in 2019.

**Region**	**Project**	**Investment ($. 000)**
Regency Jepara	158	798,950.00
Regency Batang	7	524,094.50
Semarang city	237	87,546.20
Regency Brebes	15	21,954.10
Regency Grobogan	7	17,118.80
Salatiga city	16	12,232.50
Regency Semarang	110	10,759.60
Regency Rembang	10	8,016.80
Regency Kendal	32	6,534.90
Regency Karanganyar	10	6,447.60
Regency Demak	53	4,653.00
Regency Tegal	15	2,688.50
Tegal city	8	2,363.50
Regency Pemalang	8	1,599.30
Regency Klaten	25	1,252.80
Regency Sragen	7	667.10
Regency Boyolali	31	559.50
Regency Pati	12	539.20
Regency Sukoharjo	35	523.30
Regency Kudus	7	371.80
Regency Blora	2	207.00
Regency Temanggung	10	188.70
Surakarta city	21	127.10
Regency Purbalingga	47	111.00
Regency Banyumas	26	88.00
Magelang city	6	18.10
Regency Purworejo	3	15.00
Regency Magelang	9	10.10
Regency Banjarnegara	6	8.00
Pekalongan city	8	7.30
Regency Wonogiri	7	4.10
Regency Cilacap	8	3.30
Regency Wonosobo	5	—
Total	961	1,509,660.70

Source: Dinas Penanaman Modal dan Investasi Central Java Province, 2021.

The number of sectors in the free trade era forced local governments to train their human resources, which was realized by adopting certain strategies such as improving the quality of education and honing the community’s soft and hard skills (Erliz Nindi Pratiwi, 2013; Pratiwi and Mahmudah, 2013). This is extremely important in the free market, where there are many foreign and domestic workers. However, it is feared that the influx of foreign workers will adversely affect domestic labor because they are relatively skilled compared to the local workforce.

AEC also requires Indonesian workers to have more than average skills to compete with foreign workers from neighboring countries. Therefore, there needs to be an improvement in the quality of the Indonesian workforce. The existence of AEC that triggers the mobility of goods and services indefinitely impacts the workers. This provides a large number of opportunities for the workers both at home and abroad. Therefore, the Indonesian workforce also needs to be trained to compete with other domestic and foreign workers.

## Conclusion

The opening of the free trade market led to the influx of foreign labor into Central Java Province. The existence of foreign workers has a negative or positive impact on regional economic growth. We used the Euclidean distance spatial weight matrix to calculate the spatial autoregressive model from 2015 to 2020. SAR results state that the presence of foreign labor has a positive impact on economic growth. The number of foreign workers who enter, along with the influx of investment, provides a boost for local workers to follow the performance of foreign workers.

The majority of foreign workers sent to Central Java Province are educated and skilled. The performance and work ethics of these foreign workers encourage local workers to increase their productivity. The local workforce must improve itself following the development of labor demand. Studies for local labor show that local workers have not had an encouraging impact on economic growth; this is due to the large number of local workers working in sectors that do not require high skills. Most investors who invest in Central Java Province already bring in a skilled workforce, involving only a small number of local workers. The research suggests strengthening the government’s role in increasing local labor productivity to replace skilled foreign labor. Many highlights from the industrial sector are among the limitations of the research. Other industries, such as agriculture, mining, and others, will benefit from further research.

## Data Availability

The original contributions presented in the study are included in the article/Supplementary Material; further inquiries can be directed to the corresponding author.
